# Staff perspectives on the feeding practices used in holiday clubs to promote healthy eating in disadvantaged communities

**DOI:** 10.1111/hsc.13757

**Published:** 2022-02-17

**Authors:** Natasha Bayes, Carolynne Mason, Clare E. Holley

**Affiliations:** ^1^ 5156 School of Sport, Exercise and Health Sciences Loughborough University Loughborough UK

**Keywords:** eating behaviour, feeding methods, food insecurity, healthy eating, holiday clubs, poverty, qualitative research

## Abstract

An increasing number of holiday clubs provide free meals to alleviate children's hunger during the school holidays. Holiday clubs are well‐placed to promote healthy eating among children from disadvantaged communities who may be at risk of experiencing food insecurity, but currently little is known about the feeding practices used by staff and whether these are conducive to maximising opportunities to promote healthy eating. Unlike previous research which has predominantly studied feeding practices in parent‐child dyads and childcare settings, this qualitative study explored staff perspectives on the feeding practices they use to promote healthy eating within nine UK holiday clubs working with children from disadvantaged communities. Nine individual interviews and four focus groups were completed with 27 holiday club staff during the 2019 summer holidays. Thematic analysis revealed seven feeding practice themes, including teaching about nutrition; encouraging balance and variety; modelling; involvement; non‐food rewards; restriction; and reoffering foods. The results revealed that some staff implement various positive feeding practices which align with the existing evidence‐base of feeding practices in other contexts, which is a promising finding given the current lack of information and guidance from which to draw on. However, staff also sometimes reported using maladaptive feeding practices, including overt restriction and punishment. These results emphasise the need for guidance on effective ways to implement feeding practices with children in holiday clubs. Indeed, staff demonstrated their receptivity to engaging with training resources to maximise their opportunities to promote healthy eating behaviours among children.

**What is known about this topic**

- Food insecurity is rising in the UK, and this is magnified during the school holidays when children cannot access food at school.
- Children from disadvantaged communities are more likely to experience food insecurity and have a poor diet than their affluent counterparts, which has short and long‐term adverse health and wellbeing outcomes.
- Holiday clubs provide free food during the school holidays to alleviate food insecurity and provide enrichment opportunities for children, making them a meaningful setting for promoting children's healthy eating behaviours.

**What this paper adds**

- Some holiday club staff use a variety of positive feeding practices that are conducive to promoting children's healthy eating in holiday clubs, therefore, indicating that holiday clubs have a role to play in addressing inequality.
- However, some staff reported using some maladaptive feeding practices, which should be avoided as they could negatively impact children's eating behaviours.
- Staff within holiday clubs can benefit from support including training opportunities and guidance on how to implement positive feeding practices in order to maximise opportunities to promote healthy eating and reduce inequalities.

## 1 INTRODUCTION

Holiday hunger describes families who experience or are at risk, *of seasonal hunger that occurs in households when pupils are on school holidays* (Long et al., 2021, p. 2). An increasing number of UK holiday clubs are providing free food to children to alleviate holiday hunger (Long et al., 2018). In addition to providing food, research suggests that holiday programmes such as the London‐based ‘Kitchen Social’ and North‐East England‐based ‘A day out not a handout’ have numerous benefits for children and families such as promoting health and wellbeing, and social and academic attainment (Defeyter, Stretesky, & Sattar, 2018; Defeyter, Stretesky, Sattar, & Crilley, 2018). These studies highlight that holiday clubs are a meaningful setting for enabling health‐enhancing behaviours, such as engaging children in physical activity (Graham et al., 2016) and encouraging healthy eating (Connor et al., 2015; Morgan et al., 2019). Only 18% of children aged 5–15 consume the recommended minimum daily intake of fruit and vegetables (NHS Digital, 2019), and therefore encouraging healthy eating is a major public health issue (British Medical Association (BMA), 2018). Children have an innate preference for sweet foods and aversion to sour and bitter tastes (Birch, 1999), and food preferences and aversions could explain why children scarcely consume the recommended number of fruits and vegetables (Dovey et al., 2008).

One factor known to influence children's eating behaviours is caregiver feeding practices. Feeding practices are the food‐related interactions fostered between caregiver and child, which shape the child's eating behaviour (Vereecken et al., 2010), and they can be categorised as either positive or maladaptive. Positive feeding practices encourage healthy eating behaviours in children (Kaukonen et al., 2019). Examples include modelling consumption of healthy foods, involving children in food choice and preparation, and increased availability of healthy foods (DeCosta et al., 2017). Positive feeding practices are associated with positive eating behaviour outcomes such as greater enjoyment of food and lower food fussiness among children (Holley et al., 2020). Controlling feeding practices such as pressuring children to eat and restricting food are considered maladaptive practices (Johnson & Birch, 1994). Controlling feeding practices can have counterproductive effects on children's eating behaviours as they can result in selective food preferences and reduced ability to self‐regulate energy intake (e.g. Mitchell et al., 2013).

Research related to feeding practices has predominantly been conducted with parent‐child dyads (Lumeng et al., 2012; Moens et al., 2018). Despite many children spending a significant proportion of their day in childcare settings (Hughes et al., 2007), research in these settings is limited in comparison. The foods served to children and the methods used to feed children in childcare settings shape children's food experiences and influence lifelong food habits which are established in children's early years (Briley & McAllaster, 2011; Nicklas et al., 2001). Holiday clubs targeting children from disadvantaged communities are also an important setting to explore staff feeding practices, as these children typically have a more limited diet (Morales & Berkowitz, 2016), consume fewer vegetables (The Food Foundation, 2020), and are at increased risk of experiencing food insecurity (Denney et al., 2018), developing obesity (Frongillo & Bernal, 2014), and experiencing eating disorders later in life (Hazzard et al., 2020). As feeding practices research has typically engaged more affluent communities, insights into the practices used with this at‐risk population are vital.

UK holiday clubs create multiple opportunities to promote healthy eating among child attendees (Holley et al., 2019), including providing access to a novel range of healthy foods, developing children's confidence to try new foods, and promoting positive social experiences around food. However, as no known research has been conducted which explores the feeding practices used in holiday clubs, this qualitative study sought to explore staff perspectives on the feeding practices used in holiday clubs to promote healthy eating among children from disadvantaged communities.

## 2 METHODS

### 2.1 Ethical approval

This study received ethical approval from the Loughborough University ethics committee (reference SSEHS‐2543). The lead researcher provided evidence of a full Disclosure of Barring Service (DBS) check to each participating holiday club.

### 2.2 Participants and research setting

Participants were staff delivering holiday clubs overseen by Barnardo's and StreetGames during the 2019 school summer holidays. Barnardo's and StreetGames are UK national charities that both aim to improve the lives of children from disadvantaged communities (Barnardo’s, 2021; StreetGames, 2019).

Table 1 provides a breakdown of the characteristics of the nine clubs engaged in the study, including provider name, locality, funder, guidance used to inform food provision, details of food provision, venue type, target age group, approximate number and age of child attendees. Seven were funded by the Department for Education (DfE) as part of the Holiday Activity and Food (HAF) programme, and two were non‐government funded. Clubs were located in London, Leicestershire, Newcastle and Coventry, and ran in various settings including community centres, sport centres and schools. Clubs were varied in the specific target age group and number of child attendees. Some holiday clubs were located in deprived areas while others were located in more affluent areas, but all are serving disadvantaged communities within those localities. Similarly, clubs varied in the proportion of FSM eligible children attending the club, typically varying between 50% and 90% where specific data were available.

**TABLE 1:** Holiday club characteristics

Site number	Holiday club provider	Locality	Funder	Guided by minimum standards	Food provision	Venue type	Target age group^a^	Approximate number of attendees
1	StreetGames Fit and Fed	Newcastle	DfE	Yes	Packed lunch (in‐house)	Community centre	5–12 years	10–20
2	StreetGames Fit and Fed	Newcastle	DfE	Yes	Packed lunch (outsourced)	Sport centre	6–10 years	Up to 100
3	StreetGames Fit and Fed	Newcastle	DfE	Yes	Packed lunch (outsourced)	School	Under 8 years	30–50
4	StreetGames Fit and Fed	Newcastle	DfE	Yes	Packed lunch (outsourced)	Community centre	5–11 years	Up to 70
5	Barnardo's	Leicestershire	DfE	Yes	Fresh meals prepared on site	School	3–10 years	Up to 60
6	Barnardo's	Leicestershire	DfE	Yes	Packed lunch (outsourced)	School	5–12 years	120–150
7	Barnardo's	Leicestershire	DfE	Yes	Fresh meals prepared on site	School	5–11 years	20–30
8	StreetGames Fit and Fed	Coventry	Non‐DfE	No	Fresh meals prepared on site	Community centre	7–11 years	30–40
9	StreetGames Fit and Fed	London	Non‐DfE	No	Fresh meals prepared on site	Sport centre	5–8 years; 9–13 years; 14–17 years (3 separate groups)	60–80
								

^a^ This is the age group that is targeted but children from a broader age range also attend.

Twenty‐seven club staff participated in nine interviews and four focus groups. Staff were over the age of 18 and held either a paid or voluntary role within the club.

### 2.3 Recruitment and data collection

The researcher contacted the charities Barnardo's and StreetGames to request their support in recruiting holiday clubs for the study. The charities provided a list of holiday clubs to approach for recruitment which enabled the researcher to determine whether the holiday clubs met the inclusion criteria, and to recruit a purposive sample of holiday club staff to engage in the research. The club inclusion criteria were: (1) predominantly targeted primary school aged children (e.g. age 5–11 years) consistent with the age range typically targeted in other feeding practice research; (2) primarily engaged free school meals (FSM) eligible children, where FSM eligibility serves as a reliable proxy for low socioeconomic status (Taylor, 2018) and therefore increased likelihood of experiencing holiday hunger; (3) parents not attending the clubs, as this study required insights into how staff acted as caregivers in feeding children.

The lead researcher emailed the leader of each club meeting the inclusion criteria requesting permission to approach staff to participate in the study. Willing club leaders circulated participant information sheets inviting staff to participate in an interview or focus group. Club leaders then confirmed a date for the lead researcher to visit the clubs to interview willing staff. Telephone interviews were carried out with participants who were unavailable during the visit.

Before the interviews/focus groups began, staff were asked to complete a consent form and short demographics form. Interviews lasted approximately 30–40 min, and focus groups approximately 35–65 min, with between three and nine participants in each group.

Interviews/focus groups were audio recorded and facilitated by the lead researcher using a semi‐structured approach to address the study aims while also enabling elaboration and clarification. Interview questions (Table  2) were derived from reviewing relevant literature, and through peer review from the research team.

**TABLE 2:** Interview/focus group questions

Please tell me a bit about your role(s) here in the holiday club. What are the core aspirations of your holiday club? Where is food sourced from for the holiday club provision? How do you determine what foods to serve? How do you determine when and how food is served? What methods do you use to encourage children to eat well? What does healthy food and healthy eating mean to you? How confident do you as a member of staff feel about promoting healthy eating among children? Do you promote healthy eating? What role do you feel holiday clubs can play in promoting healthy eating among children? What barriers do holiday clubs face in promoting healthy eating among children? Before we finish, is there anything else you would like to discuss relating to the topics we have been discussing today?

### 2.4 Data analysis

Interviews were transcribed verbatim and analysed, guided by Braun and Clarke's (2006) thematic analysis framework. Thematic analysis complements research positioned within an interpretivist paradigm (Braun & Clarke, 2006; Flick, 2015) and provides flexibility through the analysis process while also having the potential to generate a rich and detailed account of data (Braun & Clarke, 2006). NVivo software was utilised to enable the data to be organised into generated codes and themes.

The thematic analysis was conducted using a combined inductive and deductive approach. The inductive approach was adopted (Boyatzis, 1998) because there is no known literature available to draw upon on the use of feeding practices in holiday club contexts. A deductive approach was also adopted, drawing on the existing feeding practices evidence‐base. Other research has adopted this hybrid approach to thematic analysis (e.g. Fereday & Muir‐Cochrane, 2006).

The lead researcher (NB‐Doctoral Candidate) read each transcript to become familiarised with the data. Transcripts were coded, and from there, codes were organised into an initial thematic framework. Transcripts were further coded and recoded as the coding and thematic framework evolved. Additional researchers (CH‐Senior Lecturer, CM‐Senior Lecturer, PB‐Doctoral Candidate) performed analysis on 10% of the transcripts to ensure reliability of the analysis conducted. Team members (CH, CM) supported refinement of the thematic framework to improve its structure and robustness.

## 3 FINDINGS

### 3.1 Participant characteristics

Staff (*N* = 27) ranged from 19–59 years old (mean = 32.43 years) and were predominantly female and White British (Table 3). Most staff were educated below university level, while five (19%) had nutritional qualifications, including food hygiene qualifications, nutritional advice qualifications, and a national certificate in nutrition for sport. The number of years working at the club varied greatly, as did the staff roles.

**TABLE 3:** Participant demographic information

Demographic	*n*	%
Gender		
Female	18	67%
Male	7	26%
Missing	2	
Ethnicity		
White British	21	78%
Black/African/Caribbean/Black British	3	11%
Mixed/Multiple ethnic groups	1	4%
Missing	2	
Education		
Below university level	11	58%
Above university level	8	42%
Missing	8	
Years working in club		
<1 year	13	52%
2–5 years	6	24%
6–18 years	6	24%
Missing	2	
Staff roles		
Club leadership	8	30%
Activity leadership	7	26%
Sport and activity coaching	8	30%
Other	2	8%
Missing	2	

### 3.2 Thematic analysis

**FIGURE 1:**
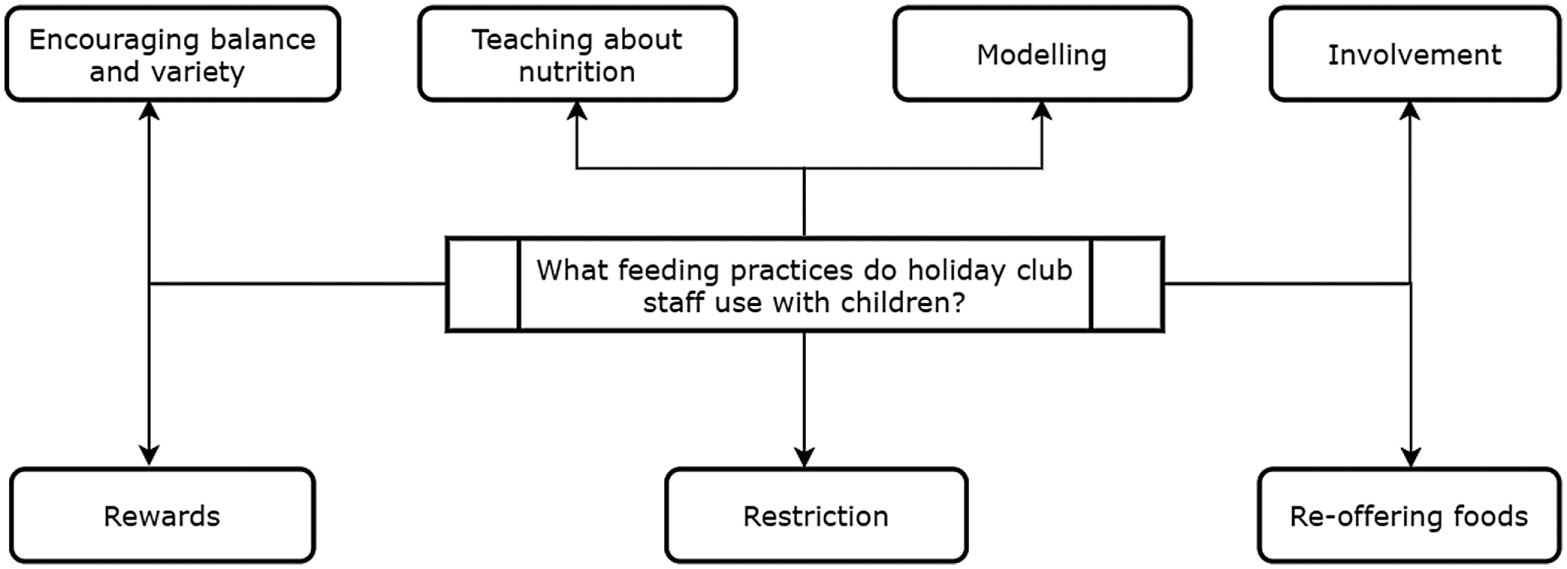
Map of feeding practice themes

Staff perspectives of the feeding practices used with children in holiday clubs were explored, revealing the use of numerous feeding practices (summarised in Figure 1). In addition, Table 4 identifies which feeding practices described by staff were positive and maladaptive feeding practices.

**TABLE 4:** Mapping the feeding practice themes to positive and maladaptive types

	Positive feeding practices	Maladaptive feeding practices
Feeding practices themes in this study	Encouraging balance and variety Teaching about nutrition Modelling Involvement Non‐food rewards Covert restriction Reoffering	Overt restriction Punishment
Implications of these feeding practices on children's eating behaviours	Associated with positive eating behaviour outcomes such as greater enjoyment of food and lower food fussiness among children (Holley et al., 2020)	Associated with negative eating behaviour outcomes such as selective food preferences and reduced ability to self‐regulate energy intake (e.g. Mitchell et al., 2013)

#### 3.2.1 Feeding practice 1: Encouraging balance and variety

Some staff discussed the importance of offering *“just a variety of things”* and “*making sure it's not the same every day,”* (INT4). This included providing foods that are healthy as well as preparing and cooking foods in ways that retain their healthy status. Additionally, staff highlighted the importance of achieving *balance between [trying to give them] something they would enjoy but then also is healthy* (INT9).

Many staff discussed offering children verbal encouragement to try new and healthy foods, such as *try it* and *dip it with this, it's nice* (INT7):

We’ll always encourage the children, 'oh I don't like that', ‘but have you tried it?’ ‘No’. ‘So try it so we can see that you've tried it’. (FG4)

Some staff highlighted a distinction between encouraging and pressuring children to eat. These staff felt that gentle, relaxed statements were likely to be encouraging while pressuring statements were often counterproductive:

The more you push it, the less likely they are to eat it, they'd be like 'why should I? Don't tell me what to do.' But if you're quite chilled about it they'd probably be willing to give it a go. (FG1)

Staff felt it was easier to encourage younger children to try new foods compared to older children and believed trying new foods from an early age may increase children's receptiveness to this later in life.

#### 3.2.2 Feeding practice 2: Teaching about nutrition

Most of the staff emphasised that holiday clubs provide opportunities to educate children about food and nutrition. Some staff believed children should learn about what food is, where it comes from and how it can impact on health and wellbeing:

I try to make them understand what it is and what they are eating and the importance of nutrition and stuff like that. (INT7)

One staff member emphasised the importance of framing such food conversations in positive ways:

…you can’t go in too heavy and tell them off because they are eating crisps or anything like that. (INT2)

Another staff member described their experience of healthy eating ‘lectures’ in school as boring and ineffective, while other staff felt that focused events and workshops specifically about food were effective strategies in teaching children about nutrition.

Many staff also felt that fun, subtle approaches such as writing poetry and songs about fruit and vegetables, and playing games, were effective ways to increase children's awareness of healthy foods:

Earlier on, we did the parachute games. Within that we played 'Fruit Bowl'. You get them to name different fruits representing the colours of the parachute, and they can play the game related to the healthy fruits they can think of. (FG3)

#### 3.2.3 Feeding practice 3: Modelling

Many staff recognised their potential to influence children as *ultimately, we are the role models, aren't we?* and that children *will take the lead from you a lot of times, won't they?* (FG3). With this understanding, staff felt that modelling encouraged positive eating behaviours in children. Staff felt that eating food with the children provided opportunities to *encourage children to do the same,* (FG2) and enabled conversations about food and sparked children's curiosity about new and healthy foods they saw staff consuming:

I've been having my raspberries with my yogurt and my nuts on top. And that's raised a lot of questions. (FG3)

Some staff highlighted that eating the same foods as children encouraged children to eat the foods provided, which was supportive when offering children healthy and unfamiliar foods. Conversely, one staff member mentioned consuming unhealthy drinks, but does so away from the children to avoid promoting unhealthy products. Some staff discussed using famous idols as models for healthy eating, including *superheroes and what they needed to be strong,* (INT9) and sport celebrities as *model examples* of healthy eating (FG3). This approach was considered particularly useful in clubs focusing heavily on sport, as sport and food are inextricably linked:

The good thing about it being sport as well is that it’s relatable to even athletes and you can use model examples of people in sports who meet those needs of healthy eating and just generally knowing what a healthy lifestyle consists of. (FG3)

#### 3.2.4 Feeding practice 4: Involvement

Many staff highlighted the importance of involving children in decisions and activities around food. Involving children in food preparation helped children to learn about food, and *because they've made it [they] are more likely to want to try it,* (FG2). Staff highlighted that while most children enjoy being involved in preparing foods, some children lack confidence because they had scarcely been involved in these activities. Involving children helped to develop their confidence, independence and skills development:

It's not that they are not engaged, it's that they think they can't do it. So they give up. You give them something to cut up and they just give up. Because they are not used to it…I think if you're a little bit tough and say 'no you need to stick this out,' by the end of it they are a lot better. (INT3)

In addition to involving children in food preparation, many staff considered it important to involve children in decisions about what foods they will eat and how much:

On the first session, we asked all the children what they would like to eat before we did a lunch. (INT3)

Many staff enabled children to choose what foods to eat from a selection, *let[ting] them have that freedom and they choose what they want,* (FG3), permitted food swaps between children and children being allowed to *save some [food] for later,* (INT1). Some staff offered second servings to children when resources permitted, but felt it was important to ensure the right portions were consumed and ensure children did not overeat:

Portion size has been hard. Because children want to eat and eat and eat. And it is hard to get that right. (INT3)

Although many staff involved children in food decisions, some staff discussed the importance of ensuring the decisions were healthy ones:

Participant 1: I mean, obviously if they say they want chocolate cake…

Participant 2: No, they can't have that.

(FG4)

#### 3.2.5 Feeding practice 5: Rewards

Using non‐food rewards was a particularly popular method used by staff to encourage children to try new and healthy foods, and to encourage children to finish their meals. Non‐food rewards took the form of stickers, certificates, raffle tickets and sporting activities. For example: *giv[ing] out raffle tickets for children who choose the healthier option,* (FG3). When giving rewards, some staff highlighted the importance of ensuring children understand why they are being rewarded:

We do the certificates and we put a reason why they got it. We don't just give them some certificate…There's a reason why they've been amazing today. (FG4)

Verbal praise was also used as a reward, which was considered an effective method for encouraging children to try healthy and unfamiliar foods:

I would walk in and they will be like ‘Oh look, look, look, salad.’ They are eating it, and I’m like ‘good boy, good girl.’ It’s a bit of a reward for them…Keep it like, whatever makes them feel good. (INT7)

However, some reward routines described by staff were more reflective of punishment, as illustrated in this quote:

So like if you don't finish this [food], you don't get to do this [activity]. (INT6)

#### 3.2.6 Feeding practice 6: Restriction

Some staff used covert restriction when feeding children, by avoiding providing unhealthy foods within the club. Some staff also used overt restriction, by telling children not to bring in unhealthy snack foods and removing sweets when they were brought in:

A young kid came in with a bag of sweets. I said to him, ‘we're not encouraging you to have that here because too much of it is not necessarily good for you…So all we will do is, we will take this, I then, when the parents come to pick you up, we will give it to your parents and they will decide what they want to do’ (INT5)

However, some staff felt that allowing small amounts of unhealthy foods was acceptable and that providing a mixture of healthy and unhealthy foods is more likely to be effective in encouraging children to eat healthy foods compared to providing healthy foods alone:

If you took away the tuck shop and just offer healthy stuff, it wouldn't work. And if you took away the healthy stuff, provided a tuck shop, you're not really doing what's best for the young people. (INT8)

#### 3.2.7 Feeding practice 7: Reoffering foods

Reoffering foods that are typically disliked by children was a strategy reported by some staff. Staff encouraged children to try foods multiple times with statements such as *it's really sweet* and as a result, children realised *this is not bad actually,* (FG4). Many staff highlighted that once children became familiar with foods, their interest and enjoyment often increased, reinforcing the benefits of persevering with exposing children to unfamiliar healthy foods. Some staff encouraged other kinds of sensory exposure, such as *have a smell first,* (FG4). Similarly, many staff recognised that children were attracted to creative food presentation, and therefore considered different ways to prepare food to increase children's enjoyment and willingness to try the foods:

…because it wasn't just an apple, it was all sliced up and it was colourful, and it was really varied, pineapples, strawberry, kiwi, grapes. I think they, yeah, they just loved it. (INT9)

Some staff recognised that exposing children to new foods is a process of trial and error, and reported that when a meal was provided and disliked, *then we can adapt that,* (FG4), highlighting staff's intention to reoffer disliked foods and adapt them to make them more acceptable:

We had some tortilla wraps. You cut them up, and we used them as crisps. But they weren't crisps…we brought them out and said, ‘who wants some crisps?’ And they were like ‘crisps?’ They were eating them and we said, ‘they are tortilla wraps.’ They were like, ‘really?’ It was just something so easy to do…It felt like a treat to them. But actually, you're eating healthy in a way. (FG4)

Similarly, some staff provide healthy foods alongside sauces and dips to encourage children to try healthy foods:

We've been offering them a lot of like, different Arab dips. So we give them that with bread sticks and some cabbage, just mix up the veggie with a bit of sauce on it, you know, little things like that. (INT7)

In addition to overtly reoffering foods, some staff hid healthy and typically disliked foods within foods that children would usually enjoy:

We had that activity didn't we? It was black bean brownies. It was kind of a really healthy, low fat brownie but they know what brownie is. (FG4)

## 4 DISCUSSION

This study explored staff perspectives on the feeding practices they use to promote healthy eating in holiday clubs working with children from disadvantaged communities. To our knowledge, this is the first study to explore the use of feeding practices in holiday club settings. The findings revealed seven themes of feeding practices used, which align with the existing evidence‐base of feeding practices among parent‐child family dyads (e.g. Moens et al., 2018), and in group contexts such as school and childcare (e.g. Elford & Brown, 2014). Most practices used were positive feeding practices, including encouraging balance and variety, teaching about nutrition, modelling healthy eating behaviours, involving children in food choice and preparation, offering non‐food rewards and reoffering foods (repeated exposure). However, sometimes maladaptive feeding practices were used, including overt restriction and punishment. Additionally, staff implemented feeding practices in various nuanced ways, resulting in typically positive feeding practices sometimes being implemented in maladaptive ways.

Staff reflected on the importance of providing healthy foods and encouraging balance and variety. This practice is supported by previous research which suggests that the increased availability of healthy foods (e.g. in the home environment) increases the likelihood of children consuming these types of foods (Campbell et al., 2007; Cullen et al., 2003), as this promotes children's healthy food choices (Nepper & Chai, 2015). Some staff also reflected their use of verbal encouragement to support children to try new and healthy foods. Encouragement and praise have been associated with increased fruit and vegetable consumption among children (Vollmer & Mobley, 2013). Staff reporting the use of these positive feeding practices provides reassuring insights that staff recognise the importance of meaningful practices that are conducive to promoting children's healthy eating behaviours.

Staff also reported using fun, subtle approaches to teach children about nutrition, such as playing games, and writing poetry and songs about vegetables. Previous research suggests using poetry during school health education lessons increases knowledge and awareness about healthy eating (Robinson et al., 2018). Similarly, staff reported involving children in food choice and preparation and using innovative approaches to modelling such as using superheroes and celebrities as models for healthy eating. Involving children in food choice and preparation is associated with greater enjoyment of food and lower food fussiness among children (Holley et al., 2020). Similarly, modelling has been associated with increased liking and consumption of fruit and vegetables (e.g. Lowe et al., 2004). The findings, therefore, highlight that staff in holiday clubs are able to implement positive feeding practices in ways that engage and appeal to children, which is important for encouraging children's healthy eating behaviours.

Staff reported using various methods when reoffering healthy and unfamiliar foods to children. For example, some staff encouraged children to try foods through sensory exposure (e.g. experiencing food through smell and sight). This technique has been found to induce short‐term decreases in food neophobia and increases in children's willingness to taste novel foods (Dazeley et al., 2012; Mustonen & Tuorila, 2010), and therefore using this technique in holiday clubs may be particularly valuable in the event that children show fussiness or reluctance to try foods being offered to them. Staff also encouraged repeated exposure by accompanying healthy foods with other foods (e.g. dips) and by hiding unpopular foods within more popular foods. Offering dip alongside vegetables has been associated with increased vegetable consumption in preschool children (Savage et al., 2013), and qualitative studies have found that caregivers often conceal vegetables within other foods (Holley et al., 2016; Pescud & Pettigrew, 2014). Importantly, staff should utilise food concealment in conjunction with serving vegetables in identifiable forms, as this increases children's awareness and liking for vegetables (Birch et al., 1998; Birch & Marlin, 1982). Finally, staff reported presenting foods in visually appealing ways to promote consumption, which aligns with previous qualitative research with caregivers (Holley et al., 2016). Although research on the effects of food presentation on children's eating behaviour is limited and equivocal (DeCosta et al., 2017; Holley et al., 2016), it is promising that holiday club staff consider the effects that food presentation may have on children's interest and willingness to try the foods provided to them.

In this study, staff revealed the use of maladaptive feeding practices, including overt restriction. Some staff described using covert restriction by avoiding providing unhealthy foods, as well as overt restriction, by telling children not to bring unhealthy foods and removing sweets from children. The overt restriction is a form of food restriction which is noticeable to the child, which can inadvertently result in restricted foods becoming more appealing and increased consumption upon availability (e.g. Ogden, 2003; Polivy & Herman, 1985). The covert restriction is therefore recommended as by making unhealthy foods unavailable, children are not explicitly denied certain foods (Ogden et al., 2006). Staff may benefit from working with parents to avoid children bringing unhealthy foods into the club, to prevent the use of overt restriction with children.

During discussions with staff about using rewards with children, some staff demonstrated using instrumental feeding (Mason, 2015), where children are rewarded for completing desired eating behaviours (e.g. finishing foods) or punished by withholding rewards when desired behaviours are not completed (Kohn, 1993; Mason, 2015; Reitman, 1988). Instrumental feeding has been associated with negative eating behaviours among children such as overeating (Rodgers et al., 2013), snacking (Rodenburg et al., 2014), and higher weight status as young adults whose parents reported using instrumental feeding when their child was young (Puhl & Schwartz, 2003). These results indicate the importance of supporting staff on effective ways to implement rewards with children to avoid the practices becoming maladaptive, such as the use of praise and encouragement for a child's willingness to taste a novel food. Nevertheless, some staff highlighted good practice with rewards, by ensuring children understand why they are being rewarded. This aligns with previous research suggesting that these verbal explanations enable children to understand and interpret feedback provided to them (e.g. Ho et al., 2015; Kohn, 1994).

The study findings show examples of both positive and maladaptive ways of implementing feeding practices with children in holiday clubs. The findings demonstrate some clear examples of good practice despite the absence of information and guidance related to feeding practices implementation, and given that only a fifth of staff has undertaken nutritional training. The findings also highlight some practices being implemented that should be avoided due to their possible negative impacts on children's eating behaviours. These results therefore highlight the need to increase awareness about what feeding practices are, and guidance on effective ways to implement feeding practices with children in holiday clubs. The provision of support resources can illustrate how staff can make simple adjustments to reframe their feeding practices from maladaptive to positive. For example, offering praise for the act of tasting foods rather than for finishing foods. Encouraging children to plate‐clear is counterproductive, as children learn that external cues (e.g. an empty plate) supersede their internal cues (e.g. feeling of fullness) (Birch et al., 1987), which reduces children's ability to self‐regulate their energy intake, and long‐term can lead to overeating (Mitchell et al., 2013).

This qualitative study was exploratory and small scale, but provides meaningful insights as the first study to explore staff perspectives on the feeding practices used in holiday clubs supporting children from disadvantaged communities, where previous research typically engages more affluent communities. This study focuses on staff perspectives of their feeding practices and does not measure their actual use of these practices. Future research utilising methods such as mealtime observations may therefore be beneficial to explore this. Additionally, research on parent's and children's perspectives of feeding practices in holiday clubs would also provide valuable insights.

It is important to highlight that the feeding practices identified in this study were not all implemented by all staff, and that staff practices were highly variable, which again indicates the value in staff accessing training and guidance to support their work. While small sample sizes are inherent in qualitative research and therefore results may not only be generalisable to the wider population (Trochim, 2021), utilising a qualitative approach enabled this study to capture rich discussions of not only what feeding practices were used, but also how staff implemented them. Staff implemented numerous feeding practices in various nuanced ways, which resulted in some typically positive feeding practices sometimes being implemented in maladaptive ways. For example, offering non‐food rewards for completing desired eating behaviours like trying new foods (a positive feeding practice) or withholding rewards if desired behaviours are not completed (a maladaptive feeding practice). This study therefore builds on the feeding practices evidence‐base, as other data collection methods such as self‐report questionnaires measure feeding practices more rigidly as either positive or maladaptive, while this study allowed the nuances and variations in their implementation to be uncovered and explored.

This study is the first to explore staff perspectives of the feeding practices they use when feeding children and promoting healthy eating in holiday clubs. Staff reported implementing a range of positive evidence‐based feeding practices in various ways which may promote positive eating behaviours in children, which is promising given the lack of feeding practices information resources and guidance from which to draw on. However, there are aspects of staff feeding practices that were maladaptive which could have negative impacts on children's eating behaviours if not appropriately reframed. These results therefore highlight the need for guidance on effective ways to implement positive feeding practices with children in holiday clubs. Indeed, staff highlighted their receptivity to engaging in training opportunities in different methods to feed children and how to implement them, suggesting staff would likely utilise such resources upon availability. The provision of support resources can illustrate how staff can make simple adjustments to reframe their feeding practices from maladaptive to positive. For example, offering praise for the act of tasting foods rather than the aim of finishing their foods. Supporting holiday club staff to promote healthy eating by enhancing their knowledge of evidence‐based feeding practices will ensure that children experiencing food insecurity are provided important opportunities to develop healthy eating behaviours. Building on this research, future research should explore what factors influence the feeding practices used in holiday clubs to identify how positive feeding practices can best be implemented in holiday clubs.

## CONFLICT OF INTEREST

There are no conflicts of interest associated with this research.

## AUTHOR CONTRIBUTIONS

All authors have substantially contributed to the design and implementation of the study as well as drafting and reviewing the manuscript. All authors have approved the final version of the manuscript and accept accountability for all aspects of the manuscript.

## ETHICAL APPROVAL

This study received ethical approval from the Loughborough University Ethics Committee (ref: SSEHS‐2543) and conforms to the ethical standards recognised by the Social Research Association “Research ethics guidance” (SRA, 2011). All study participants signed a written consent form prior to taking part in this study.

## Data Availability

Data are not available on request due to privacy/ethical restrictions (participants did not consent to data sharing).
